# Differential diagnosis of coronavirus disease 2019 from community-acquired-pneumonia by computed tomography scan and follow-up

**DOI:** 10.1186/s40249-020-00737-9

**Published:** 2020-08-26

**Authors:** Kai-Cai Liu, Ping Xu, Wei-Fu Lv, Lei Chen, Xiao-Hui Qiu, Jin-Long Yao, Jin-Feng Gu, Bo Hu, Wei Wei

**Affiliations:** 1 Infection Hospital, Anhui Provincial Hospital, the First Affiliated Hospital of USTC, Division of Life Sciences and Medicine, University of Science and Technology of China, Hefei, 230022 Anhui Province China; 2 Department of Respiratory, Hefei Second People’s Hospital, Hefei Hospital Affiliated to Anhui Medical University, 1 Guangde Road, Hefei, 230011 Anhui Province China; 3 Department of Radiology, Anhui Provincial Hospital, the First Affiliated Hospital of USTC, Division of Life Sciences and Medicine, University of Science and Technology of China, 4 Lujiang Road, Hefei, 230001 Anhui Province China; 4 Department of Radiology, Fuyang Second People’s Hospital, 450 Linquan Road, Yingzhou District, Fuyang, 236015 Anhui Province China; 5 Department of Radiology, Bo Zhou People’s Hospital, 3 Xiyi Avenue, Qiaocheng District, Bozhou, 236800 Anhui Province China; 6 Department of Radiology, Tongling People’s Hospital, 468 Bijiashan Road, Tongling, 244000 Anhui Province China; 7 Department of Radiology, Fuyang sixth People’s Hospital, 2019 Huai He Road, Fuyang, 236015 Anhui Province China

**Keywords:** Coronavirus disease 2019, Pneumonia, Computed tomography, X-ray, Differential diagnosis

## Abstract

**Objective:**

Coronavirus disease 2019 (COVID-19) is currently the most serious infectious disease in the world. An accurate diagnosis of this disease in the clinic is very important. This study aims to improve the differential ability of computed tomography (CT) to diagnose COVID-19 and other community-acquired pneumonias (CAPs) and evaluate the short-term prognosis of these patients.

**Methods:**

The clinical and imaging data of 165 COVID-19 and 118 CAP patients diagnosed in seven hospitals in Anhui Province, China from January 21 to February 28, 2020 were retrospectively analysed. The CT manifestations of the two groups were recorded and compared. A correlation analysis was used to examine the relationship between COVID-19 and age, size of lung lesions, number of involved lobes, and CT findings of patients. The factors that were helpful in diagnosing the two groups of patients were identified based on specificity and sensitivity.

**Results:**

The typical CT findings of COVID-19 are simple ground-glass opacities (GGO), GGO with consolidation or grid-like changes. The sensitivity and specificity of the combination of age, white blood cell count, and ground-glass opacity in the diagnosis of COVID-19 were 92.7 and 66.1%, respectively. Pulmonary consolidation, fibrous cords, and bronchial wall thickening were used as indicators to exclude COVID-19. The sensitivity and specificity of the combination of these findings were 78.0 and 63.6%, respectively. The follow-up results showed that 67.8% (112/165) of COVID-19 patients had abnormal changes in their lung parameters, and the severity of the pulmonary sequelae of patients over 60 years of age worsened with age.

**Conclusions:**

Age, white blood cell count and ground-glass opacity have high accuracy in the early diagnosis of COVID-19 and the differential diagnosis from CAP. Patients aged over 60 years with COVID-19 have a poor prognosis. This result provides certain significant guidance for the diagnosis and treatment of new coronavirus pneumonia.

## Background

Coronavirus disease-19 (COVID-19) is an acute infectious respiratory disease caused by severe acute respiratory syndrome coronavirus 2 (SARS-CoV-2, also called 2019-nCoV) [1]. As of June 22, 2020, in total, 8 708 008 cases have been diagnosed worldwide (http://www.chinanews.com/m/34/2020/0318/1388/globalfeiyan.html). The common symptoms of SARS-CoV-2 infection include fever, cough, shortness of breath, and dyspnoea [2–4]. In more serious cases, infection can lead to pneumonia, severe acute respiratory syndrome, renal failure, and even death. Antibodies against the new coronavirus are still being studied [5, 6], and there is no specific and effective treatment [7].

The gold standard for diagnosing COVID-19 is a positive result for the new coronavirus nucleic acid test [8, 9], but due to delays in obtaining test results or “false-negatives” in some reports, some patients cannot be diagnosed and treated in time. COVID-19 has certain imaging characteristics in the lungs. Chest computed tomography (CT) examination is an important method for the clinical diagnosis of COVID-19 [10]. The early detection of lesions is very important for early isolation and treatment and preventing the spread of the disease. Previous studies have mostly focused on the imaging manifestations and changes in COVID-19 [11–13]. The disease needs to be distinguished from many diseases by imaging, especially community-acquired pneumonia (CAP). However, comparative studies based on imaging in this area remain lacking.

The purpose of this study was to compare the clinical features and CT imaging manifestations of COVID-19 with those of community-acquired pneumonia, explore the value of CT in the diagnosis and differential diagnosis of COVID-19 and evaluate the short-term prognosis of patients with these two types of pneumonia.

## Methods

### Patient cohort

**Fig. 1:**
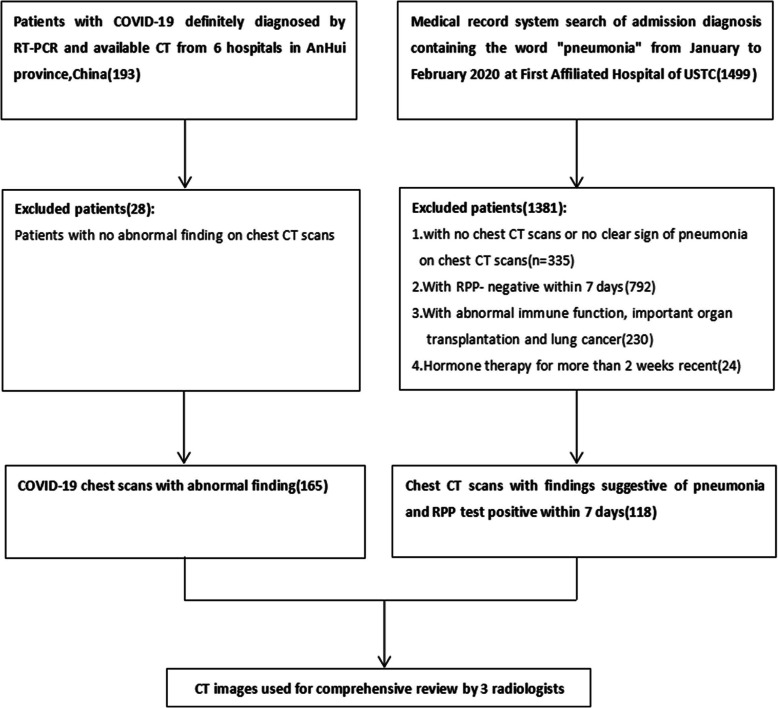
Flow diagram illustration

**Fig. 2:**
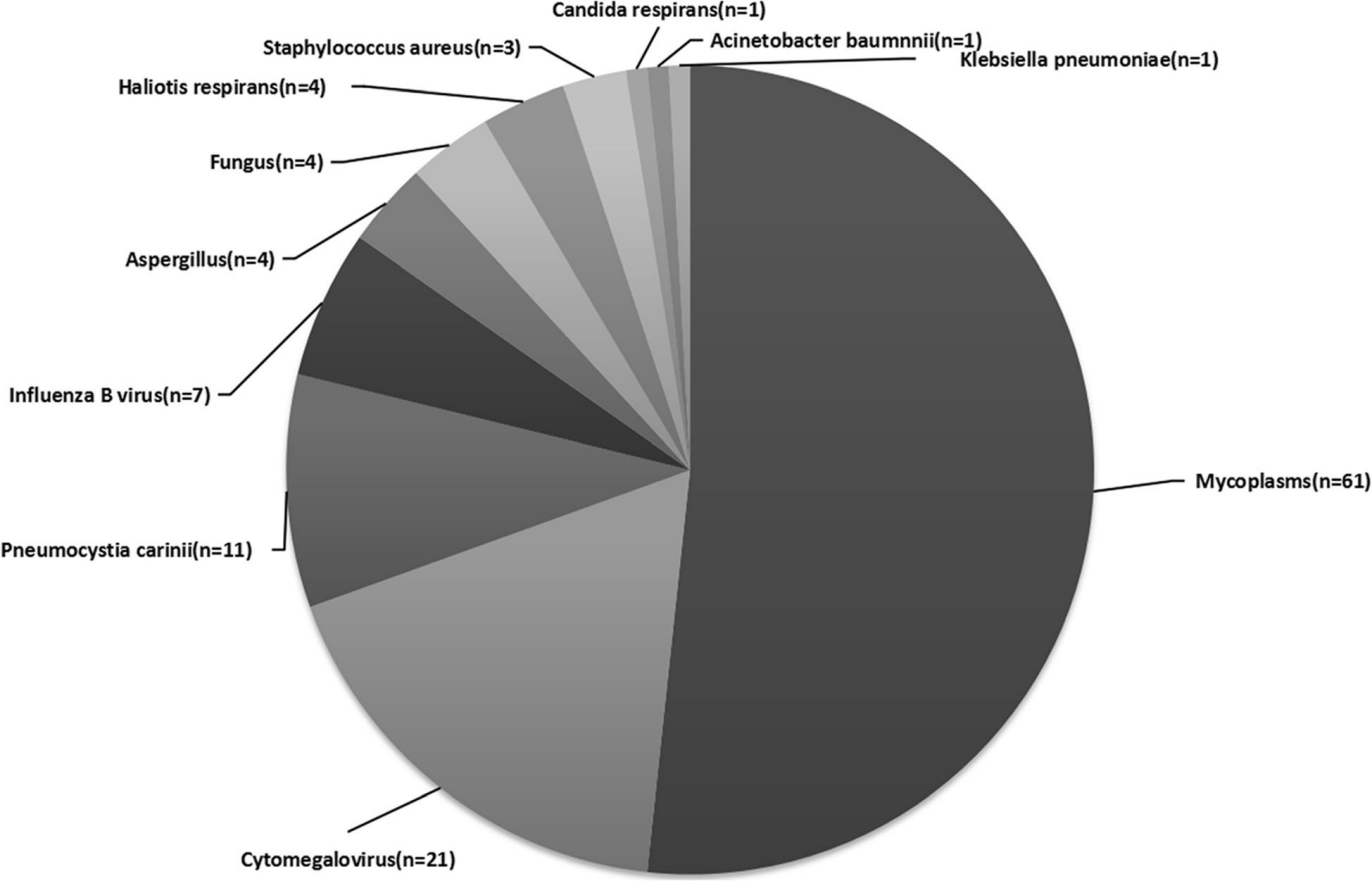
Distribution of pathogens in the community-acquired pneumonia (CAP) group based on a respiratory pathogen panel (RPP) experiment

From January 21 to February 28, 2020, the clinical data of 165 patients with COVID-19 from seven hospitals in Anhui Province, China, including 76 males and 89 females aged 5 to 91 years with an average age of 45.1 ± 17.6 years, were reviewed. The diagnosis complied with the Guidelines for the Diagnosis and Treatment of New Coronavirus Pneumonia (Seventh Edition) formulated by the National Health Committee of the People’s Republic of China [14]. In total, 118 patients with CAP were selected from the First Affiliated Hospital of the University of Science and Technology of China for pneumonia during the same period. The pathogens were confirmed in all patients by the RT-PCR detection of sputum throat swabs and blood or other pathogenic tests. The patients with CAP included 48 males and 70 females aged 1 to 76 years with an average age of 15.6 ± 21.4 years. The CT images and clinical data of all patients were collected. The inclusion and exclusion processes are shown in Fig. 1, and the results of the community-acquired pneumonia respiratory pathogen panel (CAP-RPP) are shown in Fig. 2.

### CT examination

CT scans were performed for all patients with 64-slice multidetector row CT scanners. All images were displayed and stored in the picture archiving and communication systems (PACS) system. Information regarding the CT systems in the seven hospitals is provided in Supplementary Table 1.

### CT image evaluation

The CT images of the patients in the COVID-19 and CAP groups were acquired by three experienced radiologists (who have been working in radiography for over 5 years). A double-blind method was used, and the images were read independently. The radiologists carefully observed and recorded the location, shape, number, size, edge pattern and extent of the lesion. If the diagnosis was inconsistent, a consensus was reached by the three experts.

### Statistical analysis

The measurement data are expressed as the means ± SDs, and the comparison of the groups was conducted by an independent-samples *t*-test; the count data are expressed as percentages (%), and the comparisons of the groups were conducted by the *χ*^2^ test. A correlation analysis was used to examine the relationship between COVID-19 and the age, size of the lung lesions, number of involved lobes, and CT findings of the patients. The statistical software SPSS version 22.0 (IBM Corporation, Armonk, NY, USA) was used for the statistical analysis.

## Results

### Study population characteristics

The baseline data and clinical manifestations of the two groups are shown in Table 1. The differences in age, white blood cell (WBC) count, lymphocyte proportion, and presence or absence of underlying disease in the lungs between the two groups of patients were significant (*χ*^2^ = 8.03, *P* < 0.001; *χ*^2^ = 5.58, *P* < 0.001; *t* = 4.62, *P* = 0.003; *χ*^*2*^ = 21.72, *P* < 0.001).

**Table 1:** Baseline data and clinical manifestations between COVID-19 and CAP patients

	COVID-19	CAP	*t*/*χ*^2^	*P* value
**Gender**			0.81	0.37
Male	76	48		
Female	89	70		
**Age** (years)				
Mean age	45.1 ± 17.6	15.6 ± 21.4	8.03	< 0.001
< 20	4	52		
20–40	57	8		
40–60	72	10		
> 60	32	48		
**Fever**			0.52	0.47
Yes	146	101		
No	19	17		
**Chest tightness**			0.25	0.62
Yes	31	25		
No	134	93		
**Muscle soreness**			4.76	0.03
Yes	48	21		
No	117	97		
**Fatigue**			5.11	0.02
Yes	52	23		
No	113	95		
**Cough**			2.08	0.15
Yes	85	71		
No	80	47		
**Expectoration**			25.04	< 0.001
Yes	39	62		
No	126	56		
**Headache**			1.94	0.16
Yes	10	3		
No	155	118		
**Chills**			2.76	0.10
Yes	21	8		
No	144	110		
**Diarrhea**			0.37	0.54
Yes	6	6		
No	159	112		
**WBC(**×10^9^/L**)**	9.61 ± 5.11	5.9 1 ± 3.02	4.62	< 0.001
≤ 9.5	153	67		
> 9.5	12	51		
**C-reactive protein (**mg/L)	27.81 ± 34.50	46.91 ± 56.67	1.81	0.078
≤ 8	86	14		
> 8	79	111		
**Neutrophil proportion**(%)	68.13 ± 15.28	61.69 ± 23.62	1.76	0.08
≤ 75	104	62		
> 75	61	56		
**Lymphocyte proportion**(%)	22.36 ± 12.15	46.13 ± 18.37	5.58	< 0.001
≤ 20	126	75		
> 20	39	43		
**ESR**(mm/h)	32.45 ± 24.32	57.44 ± 29.81	1.99	0.08
≤ 15	52	26		
> 15	113	92		
**Calcitonin**(mg/ml)	0.51 ± 0.69	0.39 ± 0.38	0.53	0.61
≤ 0.5	32	61		
> 0.5	133	57		
**Past medical history**				
**Heart disease**			2.37	0.12
Yes	4	7		
No	161	111		
**Hypertension**			2.67	0.10
Yes	17	20		
No	148	98		
**COPD**			21.72	< 0.001
Yes	6	25		
No	159	93		
**Diabetes**			0.06	0.81
Yes	5	3		
No	160	115		

*CAP* Community-acquired pneumonia, *ESR* Erythrocyte sedimentation rat, *WBC* White blood cell, *COPD* Chronic obstructive pulmonary disease

### CT imaging

**Fig. 3:**
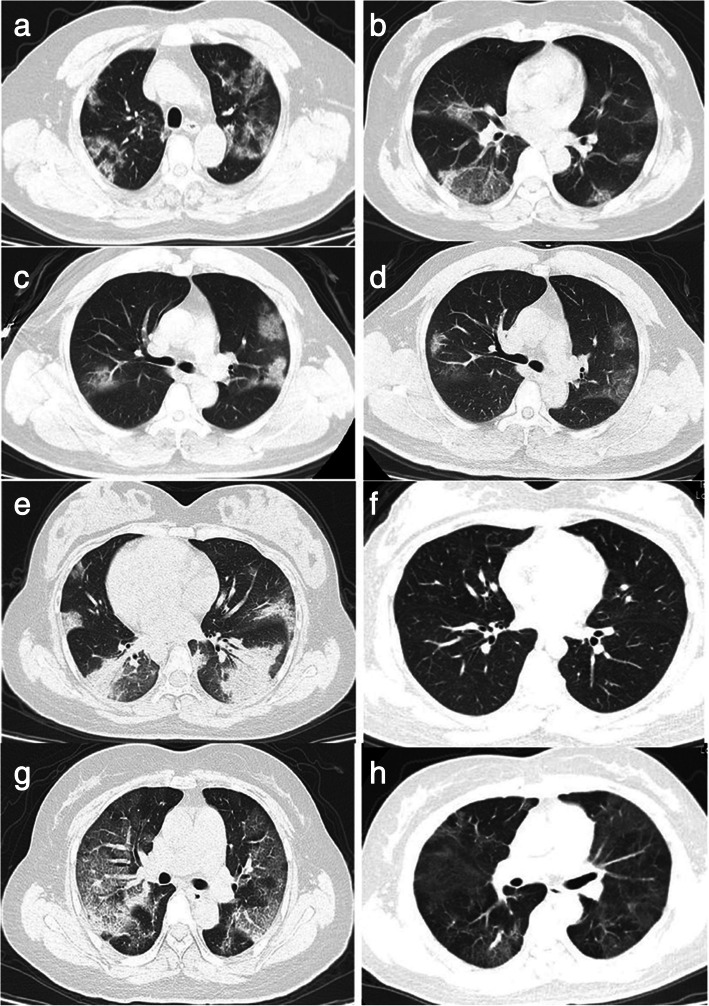
CT features of coronavirus disease-2019 patients. **a.** CT images of a 45-year-old female patient showing multiple ground-glass shadows in both lungs that were partially fused; **b.** CT images of a 42-year-old male patient showing a crazy-paving pattern under the pleura of the right lung; **c–d.** A 56-year-old female patient’s CT reviewed 7 days later showing that the original consolidation of both lungs was diminished, another ground-glass-like density shadow appeared in the middle lobe of the right lung, and the lesions in the lungs showed “wandering” characteristics; **e–f.** A 32-year-old female patient was re-examined after 10 days of treatment. CT showing that the multiple lung lesions disappeared; **g–h.** A 68-year-old female patient was re-examined after 15 days of treatment. CT showing that the diffuse lesions of both lungs were absorbed into extensive fibrosis

The chest CT findings of the patients who had COVID-19 or CAP for 3–7 days are shown in Table 2. Ground-glass opacities (GGOs) on chest CT images were found in 140 patients (84.8%) in COVID-19 group. The typical early lesions in COVID-19 group appeared as single or multiple small round lesions. In this group, 53 patients (32.1%) had small round lesions, and 87 patients (52.7%) had ground-glass-like density shadows with spots, large patches or areas of fusion (Fig. 1a). In CAP group, 47 patients (39.8%) had ground-glass shadows in the lungs, 71 patients (60.2%) showed consolidation, and the density differences between the two groups was significant (*χ*^2^ = 48.75, *P* < 0.001). In COVID-19 group, 54 cases (32.8%) exhibited a “crazy-paving” pattern mainly characterized by interlobular septal thickening (Fig. 3b) that differed from the subpleural reticular or honeycomb changes observed in the cases (28.8%) of CAP group. Eight (4.8%) patients in COVID-19 group showed “wandering” lung lesions (Fig. 3c–d).

**Table 2:** Comparison of various chest CT manifestations in COVID-19 and CAP patients

	COVID-19 *n* = 165 *n* (%)	CAP *n* = 118 *n *(%)	*χ*^2^	*P* value
**Pathological morphology and density**				
Round ground glass shadow	53(32.1%)	2(1.7%)	40.68	< 0.001
Small ground glass shadow	35(21.2%)	27(22.9%)	0.11	0.74
Large ground glass shadow	23(13.9)	13(11.0%)	0.53	0.47
Large and small mixed ground glass shadow	18(10.9%)	5(4.2%)	4.1	0.04
Ground glass shadow and solid shadow	11(6.7%)	7(5.9%)	0.20	0.66
Small patch consolidation	12(7.3%)	31(26.3%)	19.27	< 0.001
Large and small mixed patch consolidation	13(7.9%)	33(28.0%)	0.40	0.53
**Other imaging signs**				
Lesion wandering	22(13.3%)	3(2.5%)	9.05	0.002
Fibrous tissue	5(3.03%)	68(57.6%)	107.14	< 0.001
Air bronchgram	52(3.2%)	46(39.0%)	1.69	0.19
Bronchial wall thickening	18(10.9%)	37(31.4%)	18.37	< 0.001
Crazy paying pattern	54(32.7%)	34(28.8%)	0.49	0.48
Pulmonary cavity	0	12(10.2%)	17.52	< 0.001
Lung bullae	0	8(6.8%)	11.51	< 0.001
**Distribution**				
Central	17(10.3%)	22(18.6%)	4.03	0.04
Peripheral	99(60.0%)	62(52.5%)	1.56	0.21
Central + Peripheral	49(29.7%)	34(28.8%)	0.03	0.87
**Numbers of lobes and lesions involved**				
Single leaf single shot	48(29.1%)	17(14.4%)	8.38	0.03
Single leaf multiple occurrence	12(7.3%)	9(7.6%)	0.01	0.91
Multilobed multiple lesions	105(63.6%)	92(78.0%)	6.68	0.01
**Other chest diseases**				
Enlargement of heart shadow	1(0.6%)	5(4.2%)	4.37	0.04
Lymphadenopathy	1(0.6%)	18(15.3%)	23.57	< 0.001
Pleural effusion	3(1.8%)	26(22.0%)	30.57	< 0.001
Pleural thickening	2(1.2%)	31(26.3%)	41.94	< 0.001

In addition to the newly formed exudative inflammatory lesions mentioned above, the chest CT in CAP group was revealed chronic obstructive manifestations, such as emphysema, bullae, and “mosaic” signs or small airway obstructive changes. CAP group included 68 patients (57.6%) with fibrous cords or pulmonary texture aggregates, representing a significantly higher number of patients than that found in COVID-19 group (5 patients, 3.1%) (*χ*^2^ = 107.14, *P* < 0.001); bronchial wall thickening was also observed in CAP group (37 cases, 31.4%), and 18 cases (10.1%) were observed in COVID-19 group. The difference between the two groups was significant (*χ*^2^ = 18.4, *P* < 0.001). In CAP group, 12 patients (10.2%) exhibited small cavities, and 8 cases exhibited pulmonary bullae, representing a significantly higher number than the corresponding number of patients in COVID-19 group.

### Relationship between the clinical characteristics and imaging manifestations

The total number of lung lobes involved in the COVID-19 and CAP groups was 384 and 257, respectively. The number of single lesions in COVID-19 group was significantly higher than that in CAP group (*χ*^2^ = 8.38, *P* = 0.03), and the number of multi-lobar foci in CAP group was significantly higher than that in COVID-19 group (*χ*^2^ = 6.68, *P* = 0.01). The lesions in both groups were mainly located in the middle and outer lobes. In total, 148 patients (89.7%) in COVID-19 group and 62 patients (52.5%) in CAP group had lesions located in the outer zone. There was a significant difference between the two groups (*χ*^2^ = 49.6, *P* < 0.001). The statistical analysis showed that there was a positive correlation between the age of onset and the number of involved lobar segments (*r* = 0.62, *P* < 0.001).

Of the above clinical and imaging manifestations, the age factor, total number of leukocytes, and ground-glass shadows have the important clinical significance in the diagnosis of COVID-19 (Table 3). The characteristics of CAP group were consolidation in the lung lesions, accumulation of fibrous cords or textures and thickening of the bronchial wall (Table 4).

**Table 3:** Statistical prediction of some clinical and CT features for the diagnosis of COVID-19, % (*n*/*n*)

Clinical and CT features	Accuracy	Sensitivity	Specificity	Positive predictive value	Negative predictive value
20–60 years	68.9% (195/283)	59.4% (98/165)	82.2% (97/118)	82.4% (98/119)	59.1% (97/164)
WBC < 9.5 × 10^9^/L	68.2% (193/283)	91.5% (151/165)	69.5% (82/118)	80.7% (151/187)	85.4% (82/96)
GGO	62.5% (177/283)	63.6% (105/165)	61.0% (72/118)	69.5% (105/151)	54.5% (72/132)
Comprehensive	81.6% (231/283)	92.7% (153/165)	66.1% (78/118)	79.3% (153/193)	86.7% (78/90)

*WBC* White blood cell, *GGO* Ground glass opacity

**Table 4:** Statistical prediction of the diagnosis of community-acquired pneumonia by some CT signs, % (*n*/*n*)

CT features	Accuracy	Sensitivity	Specificity	Positive predictive value	Negative predictive value
Consolidation	65.7% (186/283)	60.2% (71/118)	69.7% (115/165)	58.7% (71/121)	71.0% (115/162)
Fibrous tissue	80.1% (228/283)	57.6% (68/118)	97.0% (160/165)	93.2% (68/73)	97.0% (160/165)
Bronchial wall thickness	65.0% (184/283)	31.4% (37/118)	89.1% (147/165)	67.3% (37/55)	64.5% (147/228)
Comprehensive	69.6% (197/283)	78.0% (92/118)	63.6% (105/165)	63.0% (92/146)	80.2% (105/131)

### CT follow-up of COVID-19

In total, 135 patients in COVID-19 group were re-examined 3–7 days after the first CT examination. In total, 32 patients (23.70%) showed increases in the lesion size and number and were classified as progressing, 25 patients (18.5%) were stable, and 78 patients (57.8%) were improved.

**Fig. 4:**
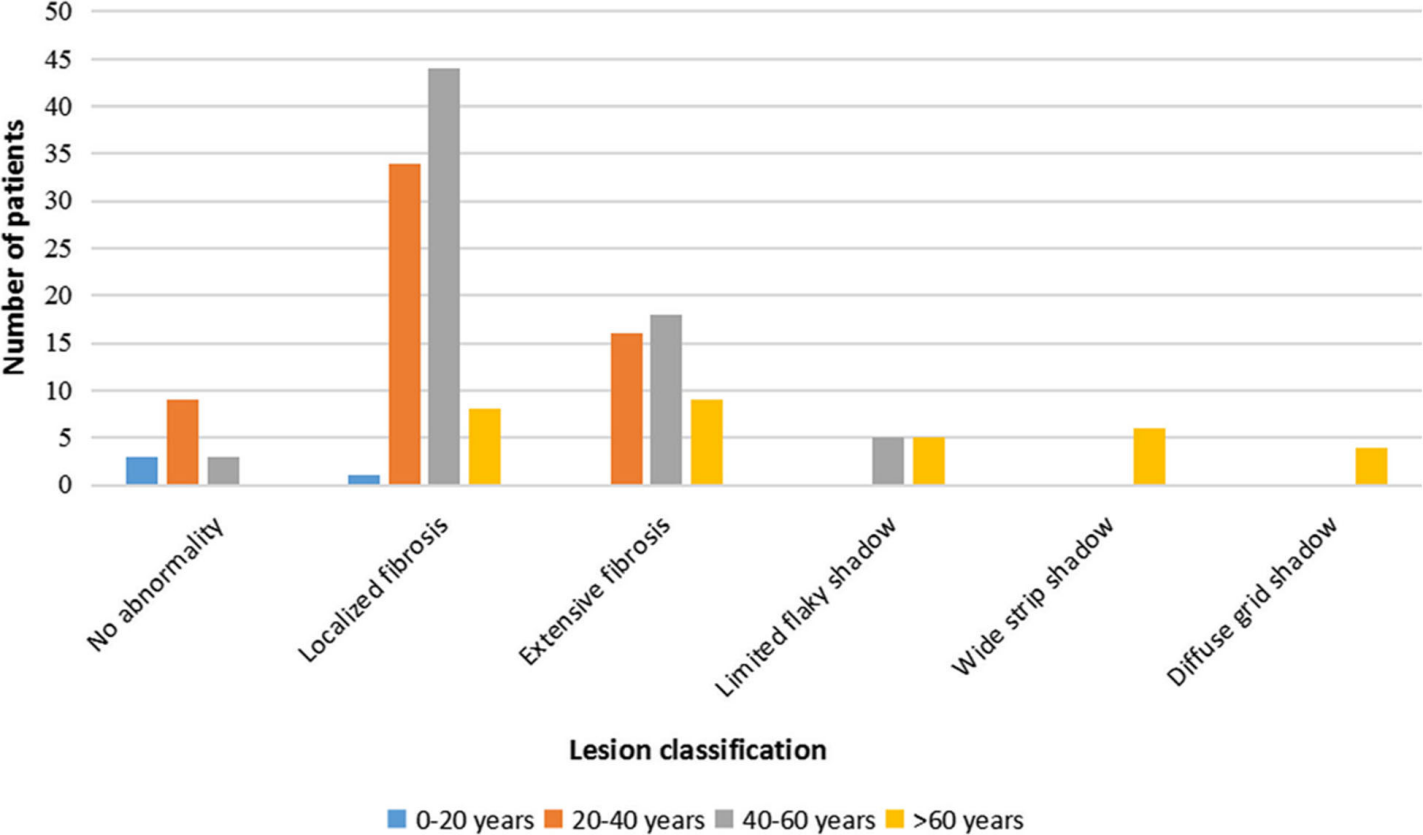
Relationship between age and prognosis in the COVID-19 patient group

All patients with COVID-19 underwent CT re-examination one month after treatment, and 15 patients (15.6%) showed no abnormalities in either lung at the time of re-examination (Fig. 3e–f). The remaining 87 patients (52.7%) showed localized strip shadows, 43 patients (26.1%) showed extensive fibrosis, 10 patients (6.1%) had limited flaky shadows, 6 patients (3.6%) had wide strip shadows, and 4 patients (2.4%) had diffuse grid shadows (Fig. 3g–h). Regarding the relationship between age and prognosis, in the patients aged < 60 years with COVID-19, the late CT changes were not significantly related to age; in the patients aged over 60 years, the CT sequelae changed with increasing age (Fig. 4 and Supplementary Table 2).

## Discussion

Currently, retrospective studies investigating various aspects of COVID-19 are being conducted. The infectivity of SARS-CoV-2 is high, the source of the pathogen is unknown, there is no specific treatment method, and the mortality rate is high [15]; therefore, the early diagnosis of COVID-19 is a challenge. A correct and timely diagnosis is of great significance for treatment and prevention of dissemination. Due to delayed laboratory results and the limited sensitivity of the tests, the controversy regarding how to obtain an early clinical diagnosis remains [16]. Thoracic imaging plays an important role in early diagnosis. We believe that in epidemic areas, diagnosis with imaging characteristics is the key to distinguish COVID-19 from other CAPs.

The data were similar to most clinical observations [17–19]. COVID-19 group mostly comprised young adults as follows: 129 patients (78.2%) were aged 20–60 years, while only 18 patients (15.3%) were aged 20–60 years in CAP group. This distribution does not comply with the general spread of infectious diseases, i.e., children and elderly populations are often the susceptible groups. Young adults typically have the strongest resistance to infectious diseases, and the incidence rate should be low. Among the 72 314 cases noted by the Chinese Center for Disease Control and Prevention, the incidence rate in China among those aged under 10 years was less than 1% [20]. The reason for the high incidence rate in young adults cannot exclude the possibility of more virus contact and infection opportunities due to more social interactions. Notably, further studies investigating the epidemiology and pathogenesis of this disease are needed. Fever was the prominent clinical manifestation in both groups, but the increase of temperature in COVID-19 group was more obvious than that in CAP group. During the early stage of the disease (within seven days), the total number of leukocytes in the observed patients with COVID-19 was normal, low (< 4 × 10^9^/L), or showed a decreasing trend, which is similar to that reported in the literature [21] and may be caused by a decrease in lymphocytes during the first few days after infection. The symptoms of muscle soreness and fatigue in COVID-19 group were significantly more prevalent than those in CAP group, while the symptoms of expectoration in CAP group were significantly more prevalent than those in COVID-19 group, which may be related to the fact that CAP is prone to producing more purulent secretion due to bacterial infection [22]. The results showed that the incidences of heart disease, hypertension, chronic lung disease and diabetes in CAP group were significantly higher than those in COVID-19 group.

Our study shows that COVID-19 chest CT reveals ground-glass shadows of different sizes, and the most typical was round or quasi circular. This shadow appeared in 32.1% (53 cases) of the patients, which is similar to other studies [13, 23]. In addition, the incidence of a single lesion in COVID-19 group was significantly higher than that in CAP group, which may be related to the early stage of SARS-CoV-2 infection. With the progression of the disease, the GGO in some patients expanded and gradually developed into ground-glass shadows with multiple patches, large areas of fusion or large and small patches coexisting, but the density change was relatively small; this finding is in contrast to the rapid consolidation observed as CAP progresses. This important feature of COVID-19 has not been reported. Notably, in some patients with mild CAP, the lung lesions did not expand until absorption. Another 32.7% (54 cases) of patients with COVID-19 showed fine reticular shadows overlapping with the ground-glass shadow, which may be related to alveolar oedema in the lung lesion area and slight thickening of the alveolar septum with infiltration of monocytes, lymph and plasma cells [24].

In CAP group, new exudative inflammatory lesions were often accompanied by obvious fibrous components or texture aggregation and cord adhesion in other parts of the lung, which manifested as bronchitis or bronchial thickening and blurring of the outer edge, suggesting that these patients had previously experienced the process of lung inflammation and that there were some remaining chronic inflammation or later changes. This finding is quite different from the acute onset, rapid progress and multiple manifestations of lung inflammation observed with COVID-19. Therefore, this study suggests that the lung findings of fibrous cord and bronchial wall thickening can be used to exclude COVID-19. In addition, a certain proportion of emphysema, pulmonary bullae, reticular or honeycomb changes under the pleura, “mosaic” signs and/or small cavity shadows in the consolidation area were observed in CAP group. In conclusion, the diversity of lung lesions and the coexistence of new and old lesions in CAP group are helpful in distinguishing CAP from COVID-19.

Six patients with COVID-19 underwent chest CT re-examinations approximately one week after the onset of the disease. The lesions showed “wandering” characteristics, which may indicate heterogeneity in the pathological changes in different lung areas; thus, early changes and changes in the organizing phase of diffuse alveolar injury can appear in different segments simultaneously, which occurs more commonly in young patients. This finding is a new finding for the diagnosis of COVID-19. However, due to the small number of patients in this group, whether these findings have a good diagnostic specificity remains to be further confirmed.

This study found that a positive correlation exists among age, the size of the lesion and total number of lung segments involved in COVID-19 group. The correlation coefficient between age and the size of the lesion was 0.522 (*P* < 0.001), and the correlation coefficient between age and the total number of lung segments involved was 0.531 (*P* < 0.001). In COVID-19 group, the elderly patients had a significantly wider range of lesions in the first visit than the young patients, and the number of involved segments in COVID-19 group was significantly higher than that in CAP group, which may be due to related reasons, such as a weak constitution and many basic diseases among elderly patients. The subsequent follow-up of COVID-19 group showed that the patients aged over 60 years had many changes remaining in the lung, and the most serious manifestation was extensive fibrous cord shadows in the lung. In this group, 32 patients (19.4%) were aged over 60 years, and the prognosis of these patients was poor. The 2- to 3-month follow-up showed that most remaining changes in the lung could be absorbed, but the absorption was slow.

This study has several limitations. First, we retrospectively analysed the imaging data of the two groups of patients. Our screening strategy has selection bias. Currently, a more balanced and large-scale prospective study involving similar patients is still needed. In this study, the different situations of the disease in each centre and the different experiences of the radiologists had some influence on the diagnosis of the disease. In addition, some patients had positive results on the new crown pneumonia nucleic acid test. However, during the winter and spring seasons, these patients often have viral and bacterial pneumonia or other diseases, which may cause some interference with the performance of CT in evaluating new crown pneumonia.

## Conclusions

In conclusion, the most common CT manifestations of COVID-19 are simple GGO, GGO with consolidation or grid-like changes. In clinical work, a CT examination should be used in combination with the epidemiological history, clinical characteristics and haematological examination to improve the accuracy of a covid-19 diagnosis and distinguish COVID-19 from CAP.

## Supplementary information

**Additional file 1: Supplementary Table 1.** Description of Chest CT protocols and parameters.

**Additional file 2: Supplementary Table 2.** The relationship between lesion classification and the number of patients in different ages(n).

## Data Availability

Additional data are available by e-mailing Kai-Cai Liu (ahsllkc@163.com) upon reasonable request.

## Abbreviations

COVID-19: Coronavirus disease 2019
CAP: Community-acquired pneumonia
ESR: Erythrocyte sedimentation rat
WBC: White blood cell
COPD: Chronic obstructive pulmonary disease
GGO: Ground-glass opacity
RT-PCR: Reverse transcription-polymerase chain reaction
USTC: University of Science and Technology of China
CT: Computed tomography
RPP: Respiratory pathogen panel

## References

[1] WHO. Novel Coronavirus – China, Jan 12 (2020) https://www.who.int/csr/don/12-january-2020-novel-coronavirus-china/en/. Accessed 19 Jan 2020.

[2] Chen N, Zhou M, Dong X, Qu JM, Gong FY, Han Y (2020). Epidemiological and clinical characteristics of 99 cases of 2019 novel coronavirus pneumonia in Wuhan, China: a descriptive study. Lancet..

[3] Wang D, Hu B, Hu C, Zhu FF, Liu X, Zhang J (2020). Clinical characteristics of 138 hospitalized patients with 2019 novel coronavirus–infected pneumonia in Wuhan, China. JAMA.

[4] Xu XW, Wu XX, Jiang XG, Xu KJ, Ying LJ, Ma CL (2020). Clinical findings in a group of patients infected with the 2019 novel coronavirus (SARS-Cov-2) outside of Wuhan, China: retrospective case series. BMJ.

[5] Bhattacharya M, Sharma AR, Patra P, Ghosh P, Sharma G, Patra BC (2020). Development of epitope-based peptide vaccine against novel coronavirus 2019 (SARS-COV-2): Immunoinformatics approach. J Med Virol.

[6] Chakraborty C, Sharma AR, Sharma G, Bhattacharya M, Lee SS (2020). SARS-CoV-2 causing pneumonia-associated respiratory disorder (COVID-19): diagnostic and proposed therapeutic options. Eur Rev Med Pharmacol Sci.

[7] Chen Y, Liu QY, Guo DY (2020). Emerging coronaviruses: genome structure, replication, and pathogenesis. J Med Virol.

[8] Zhang NR, Wang LL, Deng XQ, Liang RY, Su M, He C (2020). Recent advances in the detection of respiratory virus infection in humans. J Med Virol.

[9] Lu R, Zhao X, Li J, Niu P, Yang B, Wu H (2020). Genomic characterisation and epidemiology of 2019 novel coronavirus implications for virus origins and receptor binding. Lancet..

[10] Carotti M, Salaffi F, Sarzi-Puttini P, Agostini A, Borgheresi A, Minorati D (2020). Chest CT features of coronavirus disease 2019 (COVID-19) pneumonia: key points for radiologists. Radiol Med.

[11] Liu KC, Xu P, Lv WF, Qiu XH, Yao JL, Gu JF (2020). CT manifestations of coronavirus disease-2019: a retrospective analysis of 73 cases by disease severity. Eur J Radiol.

[12] Zhu Y, Gao ZH, Liu YL, Xu DY, Guan TM, Li ZP, Kuang JY (2020). Clinical and CT imaging features of 2019 novel coronavirus disease (COVID-19). J Inf Secur.

[13] Li X, Zeng W, Li X, Chen H, Shi L, Li X (2020). CT imaging changes of corona virus disease 2019(COVID-19): a multi-center study in Southwest China. J Transl Med.

[14] National Health Commission of the People’s Republic of China, The Guidelines for the Diagnosis and Treatment of New Coronavirus Pneumonia, seventh edition. 2020. http://www.nhc.gov.cn/xcs/zhengcwj/202003/46c9294a7dfe4cef80dc7f5912eb1989.shtml. Accessed 3 Apr 2020.

[15] Ungaro RC, Sullivan T, Colombel JF, Patel G (2020). What should gastroenterologists and patients know about COVID-19?. Clin Gastroenterol Hepatol.

[16] Lin C, Ding Y, Xie B, Sun ZJ, Li XG, Chen ZX (2020). Asymptomatic novel coronavirus pneumonia patient outside Wuhan: the value of CT images in the course of the disease. Clin Imaging.

[17] Zhou F, Yu T, Du R, Fan GH, Liu Y, Liu ZB (2020). Clinical course and risk factors for mortality of adult inpatients with COVID-19 in Wuhan, China: a retrospective cohort study. Lancet..

[18] Han R, Huang L, Jiang H, Dong J, Peng HF, Zhang DY (2020). Early clinical and CT manifestations of coronavirus disease 2019 (COVID-19) pneumonia. AJR Am J Roentgenol.

[19] Deng Y, Liu W, Liu K, Fang YY, Shang J, Zhou L (2020). Clinical characteristics of fatal and recovered cases of coronavirus disease 2019 (COVID-19) in Wuhan, China: a retrospective study. Chin Med J.

[20] Wu Z, McGoogan JM. Characteristics of and important lessons from the coronavirus disease 2019 (COVID-19) outbreak in China: summary of a report of 72 314 cases from the Chinese Center for Disease Control and Prevention. JAMA. 2020. 10.1001/jama.2020.2648. 10.1001/jama.2020.2648 32091533

[21] Huang C, Yin Y, Li X, Ren L, Zhao J, Hu Y (2020). Clinical features of patients infected with 2019 novel coronavirus in Wuhan, China. Lancet.

[22] Shi JR, Liu JR, Li HM, Wang W, Zhao SY (2016). Clinical features and therapy of persistent bacterial bronchitis in 31 children. Zhonghua Er Ke Za Zhi.

[23] Ye Z, Zhang Y, Wang Y, Huang Z, Song B (2020). Chest CT manifestations of new coronavirus disease 2019 (COVID-19): a pictorial review. Eur Radiol.

[24] Tian SF, Hu WD, Niu L, Liu H, Xu HB, Xiao SY (2020). Pulmonary pathology of early-phase 2019 novel coronavirus (COVID-19) pneumonia in two patients with lung cancer. J Thorac Oncol.

