# Supplementary material for: Differential diagnosis of coronavirus disease 2019 from community-acquired-pneumonia by computed tomography scan and follow-up
Source: Infect Dis Poverty. 2020 Aug 26;9:118. doi: 10.1186/s40249-020-00737-9 (PMC7447615; doi:10.1186/s40249-020-00737-9)
Supplement: Supplementary file 1 — Additional file 1: Supplementary Table 1. Description of Chest CT protocols and parameters. [file 40249_2020_737_MOESM1_ESM.docx]

**Supplementary Table 1. Description of Chest CT protocols and parameters**

| Hospital | CT System | Tube Volume  (kVp) | Tube Current  (mAs) | Pitch  (mm) | Matrix | Slice Thickness  (mm) | Field of view  (mm xmm) | Reconstructed Slice Thickness  (mm) |
| --- | --- | --- | --- | --- | --- | --- | --- | --- |
| Infection hospital of Anhui Provincial Hospital | Neusoft Viz-64 | 120 | 150 | 0.9 | 512 x512 | 1 | 360×360 | 1 |
| Hefei Second People's Hospital | Toshiba Aquilion-64 | 100 | 150-300 | 0.625 | 512 x512 | 1 | 350×350 | 1 |
| Anhui Provincial Hospital | Philips Brilliance-64 | 120 | 100-150 | 0.8 | 512 x512 | 1.25 | 350×350 | 1.25 |
| Fuyang Second People's Hospital | GE LightSpeed-64 | 120 | 75-150 | 1 | 512 x512 | 1.25 | 350×350 | 1.25 |
| BoZhou People's Hospital | Siemens Sensation-64 | 120 | 300 | 1.5 | 512×512 | 1 | 350×350 | 1 |
| Tongling People's Hospital | Neusoft Viz-64 | 120 | 150 | 0.9 | 512×512 | 1 | 360×360 | 1 |
| Fuyang Sixth People's Hospital | Siemens Sensation-64 | 120 | 300 | 1.5 | 512×512 | 1 | 350×350 | 1 |
