# Supplementary material for: Differential diagnosis of coronavirus disease 2019 from community-acquired-pneumonia by computed tomography scan and follow-up
Source: Infect Dis Poverty. 2020 Aug 26;9:118. doi: 10.1186/s40249-020-00737-9 (PMC7447615; doi:10.1186/s40249-020-00737-9)
Supplement: Supplementary file 2 — Additional file 2: Supplementary Table 2. The relationship between lesion classification and the number of patients in different ages(n). [file 40249_2020_737_MOESM2_ESM.docx]

**Supplementary Table 2. The relationship between lesion classification and the number of patients in different ages(n)**

| Age (year)  lesion | No abnormality | Localized fibrosis | Extensive fibrosis | Limited flaky shadow | Wide strip shadow | Diffuse grid shadow |
| --- | --- | --- | --- | --- | --- | --- |
| 0-20 years | 3 | 1 | 0 | 0 | 0 | 0 |
| 20-40 years | 9 | 34 | 16 | 0 | 0 | 0 |
| 40-60 years | 3 | 44 | 18 | 5 | 0 | 0 |
| >60 years | 0 | 8 | 9 | 5 | 6 | 4 |
